# Annotations

**Published:** 1887-12-17

**Authors:** 


### ANNOTATIONS.

**A Merry Christmas.**

WE do not very often permit ourselves to be merry in "Merrie England." Merriment seems to have been left out of our constitution. A hospital would at first sight appear to be one of the last places in the world for fun and frolic. Everything about it is suggestive of sobriety and solemnity; the beds with their sick occupants, the staid and orderly nurses, the still more staid and dignified Sisters, the grave and preoccupied physicians—all unite to give to the hospital ward a frigidity and a stiffness which are sometimes painful. The students, often mere lads fresh from school, incline a little to the infraction of this sometimes wearisome monotony; and many a sober face lights up with a smile as a well-remembered youthful voice gives kindly greeting. But even Englishmen relax once or twice a-year, and if they never, of deliberate choice, set themselves to be merry at any other time, they do so at Christmas. The hospital, too, always expects to have its full share of the genial festivities of the season. Mr. W. H. Mallock is curious to know whether or not life is worth living. Sometimes, when our fellow-mortals have been more strenuous in their antagonism than usual, we are inclined to answer in the negative. But we all try to forgive our rivals, and even our enemies, at Christmas-time, and to bring about a mental condition of general amiability to all mankind. It is our privilege and duty to ask for a specially amiable feeling towards the inmates of hospitals at the Christmas season. We say the inmates rather than the institution, because it is the former who now demand our thoughtful regard. Christmas sounds a note of goodwill to all men. Its universality is one of its strongest claims to the high place it occupies among religious festivals. The inmates of hospitals are separated from their homes and friends, and practically excluded from the busy world. Christmas decorations, Christmas fare, Christmas amusements, Christmas gifts of all kinds will now flow into the hospitals; and we may hope that an intelligent liberality will provide for the wants of the patients, old and young, men, women, and children, and so unite them for a brief period with the healthy and festive world outside. Those who are intending to send gifts should send them in the early part of the week, that they may not arrive "a day after the fair."

**The Compliments of the Season.**

"CHRISTMAS in Hospital" has in itself a sad sound. A hospital is a place where fortitude and resignation abide, but it can hardly be regarded as a congenial home for mirth, except on Mark Tapley's principle of gaining the credit of being "jolly" under difficulties. That is the task to which the hospital authorities and their volunteer helpers devote themselves at Christmas-tide. It is not an easy one, but it is well worth doing. There are few of us who have come to mature years who have not some sad memories mingled with the joyous feelings that belong to the season—few of us who have not to try to be gay. But if it requires an effort to be happy even in cheerful surroundings, how much more Tapleyan philosophy must it require to be gay when one is laid on a bed of pain, parted from our dear ones, and perhaps uncertain of seeing another Christmas on earth. And yet those who know hospital patients best will testify that the most surprising thing about the majority of them is that it takes so little to gladden them. Such little gifts, valuable only as tokens of that recognition of human brotherhood which we are so apt to forget, but which at Christmas-time is always brought before us—bright-coloured shawls and warm mittens, a few flowers, a pretty card: trifles such as these will cheer many a sad patient for more than one day. The things themselves cost little, either in money or trouble; but what gratitude and gladness they arouse! So much for the grown-up patients; but what shall we say of the children's delight at the toys and picture-books which come as a Christmas treat? Only this, that often, coming as many of the children do from homes where the festival of the Christ-child is never kept, or kept in a fashion we do not like to think of, the memory of a Christmas in hospital is so pleasant that they would fain fall ill next year about the middle of December, in order to spend Christmas there again. It is pleasant to think that, for them at least, not only private generosity, but public thoughtfulness are at work. Our contemporary *Truth* has for several years been in the habit of making a collection of toys as Christmas gifts for the children in the various metropolitan hospitals. We commend this fund to those of our readers who prefer to make such Christmas gifts in money; but the many who can more easily spare time and material may at very little cost brighten at least one day in the lives of their suffering fellow-creatures, young and old. Nothing on which a little thought and love have been expended is too trivial for such a purpose, which yet is not itself moral, but so good as to be worthy of a larger sacrifice than any we venture to ask for.

**£200 a-year for a Hospital Physician.**

The Governors of the Colchester Infirmary have decided to offer an honorarium of £200 per annum to any adventurous doctor of medicine who will consent to settle in their town and practise as a "pure" physician. They have done this apparently with the full concurrence of their medical staff. A new departure is here initiated, and a new precedent established. Persons who concern themselves with hospital affairs will be interested in seeing how the experiment will work. The first step to be taken is of course to catch the physician. Young medical men of ability and University status will hesitate before relinquishing all the possibilities of a London career for what they may consider the obscure delights of a country physician's life. But what does it matter where a man spends his life, provided he spends it in good work well done? Colchester is a country town, and can never offer to any man those rewards which are looked upon as the highest in the profession. But it can probably offer a career worthy of a capable man's consideration, who is animated by the highest ideals of the physician. There is something to be said in favour of a post which will be admittedly the highest that Colchester medicine has to offer. Some men would rather be first in Colchester than sixth, or twelfth, or fiftieth in London. An active and energetic man, too, might exercise a highly stimulating influence on the profession at Colchester and in the surrounding districts. He would be under no temptation to enter into unwholesome rivalry with his brother practitioners. He would have time on his hands and large opportunities of study and practical work. The infirmary contains a hundred beds, nearly half of which will probably be occupied by medical cases. How few London physicians can have charge of so many in-patients? The Governors at Colchester have acted with a good deal of public spirit; and it is much to be desired that a physician may be found who will justify their desire to maintain the best traditions of medicine in the town. The medical staff, too, seem to be thoroughly in accord with the Governors, and that is an indication of much promise to any man who may be turning his eyes to country practice. It may be hoped that the experiment now to be made will not be spoilt by the selection of a man of inferior capacity and education, solely on the strength of social advantages or particular degrees. What is wanted is a man of really independent mind, honourable character, and proved ability, who will practise his art on the broadest lines, and make it a name of honour and respect in the town and county. It is too much the fashion among hospitals to select men solely on account of their academic status. Medicine is the only profession in the world where such a course is possible. In the Church and at the Bar proved capacity is essential in those who aspire to fill high places. When will it be so in the hospital world?

